# From rivers to the sea: a single gene assay for New Zealand freshwater eel eDNA monitoring

**DOI:** 10.7717/peerj.21612

**Published:** 2026-08-05

**Authors:** Therese C. Miller, Xavier Pochon, Alexandre Che-Pelicier, Hannah Hampton, Michelle Scriver, Amandine Sabadel, Julia M.I. Barth, Chrysoula Gubili, Robert Jehle, Michael Matschiner, Ezgi Ogutcen, Robert Schabetsberger, Emily C. Giles

**Affiliations:** 1 Institute of Marine Science, University of Auckland, Auckland, New Zealand; 2 Cawthron Institute, Nelson, New Zealand; 3 School of Science, Auckland University of Technology, Auckland, New Zealand; 4 Sequench Ltd., Nelson, New Zealand; 5 Department of Environmental Sciences, Zoological Institute, University of Basel, Basel, Switzerland; 6 Faculty of Biology, Ludwig Maximilian University of Munich, Planegg-Martinsried, Germany; 7 Fisheries Research Institute, Hellenic Agricultural Organisation-DIMITRA, Kavala, Greece; 8 School of Science, Engineering and Environment, University of Salford, Salford, United Kingdom; 9 Natural History Museum, University of Oslo, Oslo, Norway; 10 Institut National de la Recherche pour l’Agriculture, l’Alimentation et l’Environnement (INRAE), Villeurbanne, France; 11 Department of Environment and Biodiversity, University of Salzburg, Salzburg, Austria

**Keywords:** *Anguilla dieffenbachii*, *Anguilla australis*, Droplet digital PCR, Environmental DNA, NADH5

## Abstract

Use of environmental DNA and RNA (eDNA and eRNA) is rapidly expanding as an unintrusive and timely method of studying rare or cryptic organisms. These methods have been proposed for studying the unknown migration routes and spawning sites of Aotearoa New Zealand longfin (*Anguilla dieffenbachii*) and shortfin (*Anguilla australis*) eels. However, these areas may be in sympatry with other members of the genus, necessitating species-specific eDNA and eRNA assays. Accurately comparing eDNA and eRNA quantity and degradation rates of two different species can only be done when assessed on the same gene. In this study, species-specific droplet digital polymerase chain reaction (ddPCR) primers and probes were developed for two closely-related species of catadromous eels, *A. dieffenbachii* and *A. australis*. Primer and probe assays were created using phylogenetic inference and novel software (assayID) to identify regions of the mitochondrial genome that remain conserved within species but are sufficiently differentiated between target species and other congenerics. In consensus trees, non-anguillid outgroups clustered separately from the Pacific anguillid taxa for the marker of interest, indicating that there is considerable divergence between the assay region in anguillids compared to other taxa. Assays were designed targeting the same region of the nicotinamide adenine dinucleotide hydride 5 (NADH5) mitochondrial gene. The targeted regions span 174 bp for *A. dieffenbachii* and 177 bp for *A. australis*. Primers were tested against other species of Pacific *Anguilla* in end-point PCR, and probes were evaluated in ddPCR. Although faint bands of non-target amplification were observed in end-point polymerase chain reaction (PCR), ddPCR showed minimal cross-reactivity. A ten-fold dilution series of target species’ genomic DNA was used in a multiplex reaction with these primers and probes to assess limits of detection and quantification (LOD and LOQ). Both assays achieved high sensitivy with LOD and LOQ values of 0.20–0.51 copies/µL. Degradation rates of *A. dieffenbachii* and *A. australis* eDNA and eRNA were quantified by applying the developed ddPCR assays to a previously generated set of samples from a controlled in-tank degradation experiment conducted over 168 hours at nine time points. The eDNA half-life for both species ranged from 5 to 23 hours, and the eRNA half-life ranged from 4.5 to 16 hours. For both species, greater amounts of eDNA were detected than eRNA. For both assays, strong associations were found between the concentration of loaded DNA and ddPCR copy numbers. Future use of these assays on collections from the wild will further validate their utility in diverse environmental and sympatric contexts. These assays establish a reliable framework for species-specific detection of *A. dieffenbachii* and *A. australis* where multiple *Anguilla* species may be present and hold potential to unveil their migration routes and cryptic spawning areas.

## Introduction

The advent of environmental genetics has enabled fast and scalable detection of organisms, which is important for understanding ecosystems in a changing world. Molecular genetic tools are proving to be highly effective for recording local species dynamics where traditional methods, such as observations, satellite tags and sampling surveys, are challenging and costly (Higuchi et al., 2021; Koster et al., 2021; Doi et al., 2021). Additionally, genetic tools allow for distinction between closely related taxa where morphological identifications have previously been inconclusive (Watanabe et al., 2013; Boykin & De Barro, 2014). Environmental DNA (eDNA) and environmental RNA (eRNA) are highly valuable tools for monitoring biodiversity in a range of environments (Iacaruso et al., 2025). Quantitative Polymerase Chain Reaction (qPCR) and droplet digital PCR (ddPCR) are increasing in use for species-specific detection of rare taxa (Moyer et al., 2023; George et al., 2023; Li et al., 2023). These techniques offer high species specificity and can reduce masking by highly abundant taxa, thereby improving detection of low-abundance targets (Wood et al., 2019). Droplet digital PCR also has greater sensitivity than qPCR, as every ddPCR reaction is the equivalent to roughly 20,000 end-point PCR reactions (Doi et al., 2015), is less impacted by inhibitors than qPCR (Sidstedt, Rådström & Hedman, 2020), and lacks many of the nuances that qPCR requires, such as triplicate sample testing or addition of inhibition assays (Wood et al., 2019; Lewin et al., 2020). Consequently, ddPCR is generally considered as a highly reliable and sensitive technique for species-specific detection, particularly in complex environmental samples (Guri et al., 2024).

Across the Atlantic, Pacific, and Indian Oceans, *Anguilla* species comprise important commercial fisheries that, until the 1970’s, totalled thousands of tons of catch (McDowall, 1990; Casselman, 2003; Tatsukawa, 2003). They are still extensively harvested and cultured today, particularly in east Asia (Yuan et al., 2022). Research efforts to understand and conserve *Anguilla* eels have increased over the last 20 years due to startling declines in population sizes because of a wide array of anthropogenic influences (Miller et al., 2026). Despite their population declines, global fishing and trading of freshwater eels has been ongoing, even for species that are considered endangered or data deficient (Stein et al., 2016; Stein et al., 2021). This highlights the need not only to detect *Anguilla* products in the market, but also to make environmental assessments to determine the natural ranges and population sizes of these iconic fishes. Uncovering the cryptic migration routes and unknown spawning areas of these species can allow managers to make better informed decisions on where to target conservation efforts.

In Aotearoa New Zealand (hereafter NZ), freshwater eels (genus: *Anguilla*) are of considerable cultural and economic importance (Jellyman & Todd, 1982). New Zealand has three native species of freshwater eels: the longfin eel *A. dieffenbachii*, the shortfin eel *A. australis*, and the speckled longfin eel *A. reinhardtii* (Jellyman et al., 1996). *Anguilla dieffenbachii* is endemic to NZ, is considered endangered and has an unknown population trajectory (IUCN http://www.iucnredlist.org). *Anguilla reinhardtii* is listed as least concern, and *A. australis*, including its two recognized sub-species *A. australis australis*, and *A. australis schmidtii*, is considered near threatened by the IUCN. Spawning areas for these three species are theorized to be in the south Pacific Ocean (Miller, 2023) where ten other species of *Anguilla* eels are also thought to spawn. Thus, these species are likely to experience sympatric life stages with other members of their genus (Kuroki et al., 2020; Miller, 2023). These nuances of the biology of Pacific eels highlight the need for eDNA assays that are highly species-specific.

Considerable effort has gone into developing species-specific assays to detect both *A. dieffenbachii* and *A. australis* in NZ freshwater systems (Thomson-Laing et al., 2021; Thomson-Laing et al., 2022; Thomson-Laing et al., 2023). The existing *A. dieffenbachii* detection assay targets mitochondrial *cytochrome b* (CytB), and the *A. australis* assay targets mitochondrial 16S rRNA (16S) (Thomson-Laing et al., 2021). By targeting different genes, these assays reliably distinguish these two species from each other, from co-occurring freshwater fish in NZ, and are also appropriate for primer and probe assay design (Bustin, Mueller & Nolan, 2020). However, the design of eDNA and eRNA markers on separate genes complicates comparative estimates of relative abundance. The assays of NZ freshwater eels were used in a controlled experiment to calculate the concentrations and decay rates of *A. dieffenbachii* and *A. australis* eNAs (Che-Pelicier et al., 2025). Differences in detecting shed amounts of eNAs and differential decay rates may have been due to examining different species, or they could have been due to targeting different mitochondrial genes. To accurately ascertain the reason for differences in detection of eNAs and their decay rates, examining the eNAs of these two species on the same mitochondrial gene is necessary.

The objective of this study was to test the limits of a single-gene ddPCR approach for reliably discriminating closely related anguillid eels with overlapping distribution ranges. We hypothesized that in-silico design of species-specific primers and TaqMan probes would allow us to (i) optimize the selection of a single-mitochondrial locus to target both species of interest while (ii) avoiding cross-reactivity with other anguillid non-target species, and (iii) ensure that both assays achieve sensitivity and precision suitable for low-concentration eNA detection. This was assessed from empirical validation, quantifying specificity, analytical sensitivity, and repeatability under standardized ddPCR conditions. Overall, the design of single-gene assays for *A. dieffenbachii* and *A. australis* was optimized using comparative analyses of complete mitochondrial genomes from multiple individuals across 13 *Anguilla* species. These assays are powerful molecular tools for species-level detection of NZ freshwater eels for future ecological and monitoring applications.

## Materials & Methods

### Primer and probe design

Primers and internal probes were designed to identify and differentiate between two target eel species (*A. dieffenbachii* and *A. australis*) while assaying the same mitochondrial gene. For this, mitochondrial protein coding sequences were retrieved from NCBI (see Table S1 for accessions) for initial phylogenetic inference. Afterwards, 344 whole mitochondrial genomes of all known species of *Anguilla*, extracted from a larger genomic dataset (JMI Barth, M Matschiner, R Schabetsberger, 2025, unpublished) and assembled with the programs MITObim (v.1.8; Hahn, Bachmann & Chevreux, 2013) and MIRA (v.4.0.2; Chevreux, Wetter & Suhai, 1999), as described in Ronco et al. (2021) were used for assay design. The 344 genomes provided were generated as part of the project (10.55776/P34091), funded by the Austrian Science Fund (FWF).

**Figure 1:**
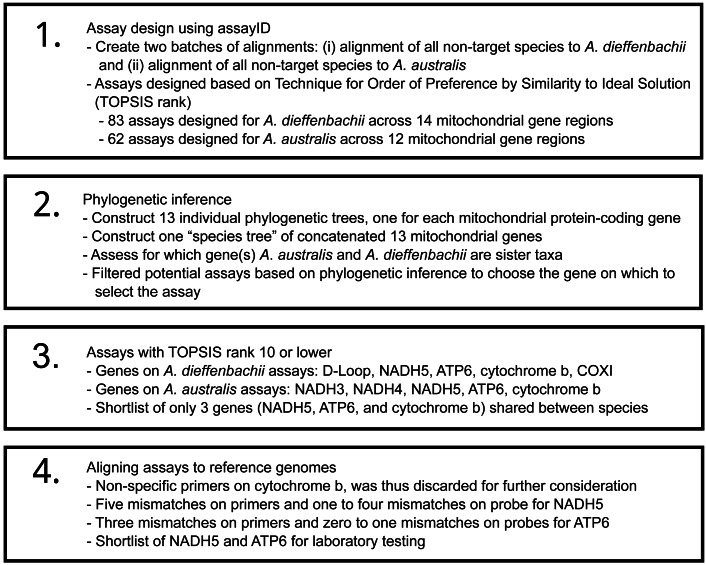
The process of screening mitochondrial genes for identification. The process followed to design species-specific assays on the same mitochondrial gene for *A. dieffenbachii* and *A. australis*.

Assay design was conducted using phylogenetic inference and by comparing full mitochondrial genomes of multiple individuals of anguillid eels (Fig. 1). Note that, during in-silico primer and probe design, multiple mitochondrial genomes of each species were included in the analyses. These included multiple individuals of both subspecies of *A. australis*. For the purpose of primer and probe design, we refer only to the species level, *A. australis*. Regions of the mitochondrial genome with high sensitivity (*i.e.,* low intra-specific variability) and high specificity (high genetic distance between target and non-target species) were identified using assayID (https://github.com/jammc313/assayID). All mitochondrial genomes with the exception of the second target species were used as inputs. For example, when *A. dieffenbachii* was the target species, *A. australis* was withheld from the analysis whilst searching for potential primer regions that differed between *A. dieffenbachii* and the outgroup species. The rationale for withholding the second target species from the initial design was based on (i) the restriction of assayID to only one target species and (ii) to avoid excluding gene regions of moderate similarity between target species. Here, it was important to design an assay that is similar enough among target species to be included on the same gene while also being differentiated enough to distinguish between target species. The in-silico assays were ranked by Technique for Order of Preference by Similarity to Ideal Solution (TOPSIS Rank; https://github.com/jammc313/assayID) which is based on a multivariate statistical method taking into account greatest interspecific genetic distance between target and non-target species, least intraspecific genetic distance within target species, guanine-cytosine (GC) content, and absence of hairpins. Primers with the ten highest rankings for each target species were retained as candidate primers. These were then aligned to the annotated mitochondrial genomes (alignment function; https://www.geneious.com) in Geneious Prime (Kearse et al., 2012) to identify the mitochondrial gene for which the assays were designed. The candidate primers were further filtered to only include those designed for a given mitochondrial gene for both target species. Then, the consensus alignments of the filtered candidate assays were aligned for both target species and the number of mismatches in the primer and probe regions were tallied to guide final assay selection.

To corroborate the resolution of the single mitochondrial genes for discerning between the closely-related targets and other pelagic eels, phylogenetic trees were built using the mitochondrial protein-coding genes for the filtered candidate assays. Sequences for the assay genes were downloaded from NCBI (see Table S1 for accessions). Each tree was built using the best-fitting substitution model (modelfinder; Kalyaanamoorthy et al., 2017) and a maximum likelihood framework. Branch support and consensus tree topology were determined using 1,000 bootstrap replicates (IQ-TREE, Nguyen et al., 2015). RStudio version 4.5.1 was used to improve the visualization of the phylogenies using ape (5.8.1; Paradis & Schliep, 2019), phytools (2.4.4; Revell, 2012), and ggtree (3.12.0; Yu, 2022). The criteria for inclusion of a candidate assay was a TOPSIS rank of 10 or less, sharing of the same mitochondrial gene for both target species, inclusion of base pair mismatches in primers, and the ability to resolve the *A. australis* subspecies and *A. dieffenbachii* as sister taxa.

### DNA extractions

Tissue samples of *Anguilla* specimens either frozen or stored in RNAlater were used for nucleic acid extractions. Tissue samples were extracted from *A. bengalensis*, *A. reinhardtii*, *A. marmorata*, *A. megastoma*, *A. bicolor*, *A. celebesensis*, *A. luzonensis*, *A. obscura*, *A. interioris*, *A. japonica*, *A. australis*, and *A. dieffenbachii*. Permits for obtaining the eel tissues are described in Barth et al. (2020). To remove RNAlater from the tissue samples, samples were spun at 10,000 g for 60 s and dabbed dry with sterilized KimTech wipes before transferring to 1.5 mL elution tubes. DNA was extracted from eel tissues using the DNEasy Blood and Tissue kit (Qiagen) following the manufacturer’s protocol with a 56 °C incubation for three hours. One negative control was included in each extraction run using only extraction kit buffers and no tissue sample (extraction blank). The concentration of each extraction was checked using a NanoPhotometer (Implen) and a Qubit dsDNA high sensitivity kit (ThermoFisher Scientific). Before and after conducting DNA extractions, the workspace was left under UV light for 30 min. Tweezers were sterilized between handling each sample by dipping them in 10% bleach followed by sodium thiosulfate and then dipped in ethanol and held over a flame.

### End-point polymerase chain reaction

Before and after conducting PCR, all pipette tips, pipettes, PCR tubes, and water were left under UV light for 15 min. All work surfaces were sterilized using 10% bleach and then 70% ethanol. PCR preparations were conducted under a laminar flow cabinet with HEPA filtration. The NADH5 primers of *A. dieffenbachii* (LF_NADH5_F and LF_NADH5_R), the NADH5 primers of *A. australis* (SF_NADH5_F and SF_NADH5_R), the ATP6 primers for *A. dieffenbachii* (LF_ATP6_F and LF_ATP6_R) and the ATP6 primers for *A. australis* (SF_ATP6_F and SF_ATP6_R) were tested initially using end-point polymerase chain reaction (PCR) (Table 1). Species-specific assays included DNA extractions from tissue and eDNA samples of filtered water from two specimens of each target species (*A. dieffenbachii* or *A. australis*), as well as DNA from one tissue specimen of each non-target species (*A. megastoma*, *A. obscura*, *A. marmorata*, *A. celebesensis*, *A. luzonensis*, *A. bengalensis*, *A. reinhardtii*, *A. bicolor*, *A. japonica*, and *A. interioris*). To confirm extracted DNA quality of genomic DNA, PCR was conducted using MiEel primers following the protocol in Takeuchi et al. (2019) and then PCR products were run on a 1% agarose gel at 100 V for 30 min. After successful amplification using MiEel primers, a PCR assay was designed using a gradient PCR and the primers designed in this study (LF_NADH5_F, LF_NADH5_R, SF_NADH5_F, SF_NADH5_R, LF_ATP6_F, LF_ATP6_R, SF_ATP6_F, and SF_ATP6_R). Annealing temperatures (Ta) from 52 °C to 60 °C were evaluated, and PCR products were checked on a 1% agarose gel. The PCR parameters were optimized for high target amplification and minimal nonspecific binding. Each PCR reaction had a total of 30 µL consisting of 15 µL of 2x MyFi mastermix (Bioline, UK), 11 µL of deionized water, 1 µL of 10 µM of each forward and reverse primer, and 2 µL of DNA at a concentration of 10 ng/µL. A negative control containing only mastermix, water, and primers (no template control; NTC) was included in each PCR reaction. The PCR cycling conditions are as follows: hold at 95 °C for 2 min, 35 to 40 cycles of 95 °C for 20 s, gradient annealing temperatures for 20 s, 72 °C for 20 s, and a final hold at 72 °C for 10 min. The PCR protocol was refined by choosing the highest annealing temperature (60 °C) and then reducing the number of cycles (35).

**Table 1:** Tested primer/probe assays designed by assayID.

Target species	Target size (bp)	Primers/Probe	Sequence
*Anguilla dieffenbachii*	174	LF_NADH5_F	5′—TCT TTG CCG CCA TAG AGC TA—3′
		LF_NADH5_R	5′—GAT CTA CTA ATT GGG TGG CTA CT—3′
		LF_NADH5_Probe	5′-/5HEX/ACT TCC CAG /ZEN/CCG TAA TCC ACC GAA TAG CCC /3IABkFQ/-3′
	108	LF_ATP6_F	5′—GTC CCT CTA ATC CCT GTG CTA—3
		LF_ATP6_R	5′—TAA GAG ATG GCC TGC TGT CA—3′
		LF_ATP6_Probe	5′—TCC GTC CAC TAG CCC TAG GTG TAC GAC TCA —3′
*Anguilla australis*	177	SF_NADH5_F	5′—GAC TCC TCA CCG CCA TAG AA—3′
		SF_NADH5_R	5′—GGT CTA CTA ATT GGG TGG CTA CT—3′
		SF_NADH5_Probe	5′-/56-FAM/CTT CCC AGC /ZEN/CGT AGT TCA CCG AAT GAC CC/3IABkFQ/-3′
	171	SF_ATP6_F	5′—AAC CAA CCG TAG CAC TAG GA—3′
		SF_ATP6_R	5′—CGG TGG CGA TGA GTT GAA TT—3′
		SF_ATP6_Probe	5′—CAC CTA CTG CCA GAA GGA ACA CCA GTC CCT—3′

### Droplet digital PCR species specificity

The NADH5 (LF_NADH5_Probe) and ATP6 (LF_ATP6_Probe) probes for *A. dieffenbachii* as well as the NADH5 (SF_NADH5_Probe) and the ATP6 (SF_ATP6_Probe) probes of *A. australis* were tested in singleplex assays along with their respective primers to assess for cross-species amplification in ddPCR (Table 1). The probes for *A. australis* NADH5 and ATP6 genes were dual-labelled with a 5′ 6-carboxyfluorescein (6-FAM) fluorescent tag and a 3′ Iowa Black (IB) fluorescent quencher (FQ), and the *A. dieffenbachii* probes for both NADH5 and ATP6 genes had a 5′ hexachlorofluorescein (HEX) fluorescent tag and a 3′ IB FQ. These singleplex assays included DNA from extracted tissue samples of *A. dieffenbachii* and *A. australis*, eDNA samples of *A. dieffenbachii* and *A. australis* held in controlled conditions generated by Che-Pelicier et al. (2025), and tissue samples of other species of *Anguilla* that spawn in the Pacific Ocean. Concentrations of all tissue samples in these assays were tested at 1 ng/µL and 0.1 ng/µL. Each 20 µL ddPCR reaction consisted of 10 µL of 2x ddPCR Supermix for Probes (No dUTP; Bio-Rad), 3 µL ddH2O, 1 µL of each 10 µM forward primer, 1 µL of each 10 µM reverse primer, 1 µL of each 10 µM probe, and 1 µL of loaded DNA. Every ddPCR run contained a NTC. The ddPCR mixture was combined with 70 µL of oil for probes by the BioRad QX200 ddPCR droplet generator (Bio-Rad). Twenty microliters of the resulting nanodroplet emulsion was deposited into a 96-well plate, and then PCR was conducted in a BioRad C1000 Touch Thermal Cycler (Bio-Rad) using the following conditions: 95 °C for 10 min for initial denaturation, 40 cycles of 94 °C for 30 s and 60 °C for 1 min, and a final step of 98 °C held at 10 min for enzyme deactivation. Droplets were read and analyzed on a BioRad QX200 droplet reader (Bio-Rad) and visualized using QuantaSoft Analysis Pro (1.0.596). Thresholds were manually set (10,000 for *A. australis* and 4,000 for *A. dieffenbachii*) based on amplitudes of positive and negative droplets.

### Limits of detection and limits of quantification

A duplex ddPCR reaction was then conducted to test efficiency of the assays in a seven-fold series of 10 × dilutions. These included DNA extracted from tissues of both target species *A. dieffenbachii* and *A. australis*. Sample concentrations ranged from 10 ng/µL to 0.01 pg/µL. Starting concentrations of loaded DNA began at 10 ng/µL because higher concentrations displayed oversaturation and inhibition effects (“rain”, Porco et al., 2022). This assay was run in six technical replicates and included a PCR NTC for negative controls. The limit of detection (LOD) was calculated based on the lowest level of detection that was greater than the maximum value of the negative controls (Baker et al., 2018). The limit of quantification (LOQ) was calculated based on the lowest detected concentration with a coefficient of variation (CV) value above 35% (Klymus et al., 2020). Exponential regressions were performed using the nls function in R and the stats package (R Core Team, 2025) to correlate amount of loaded DNA and copy numbers of amplicons detected for each species. All statistical analyses were conducted in R−4.5.1 (R Core Team, 2025).

### Degradation rates

To calculate the rate of eDNA and eRNA degradation for the gene of interest in *A. australis* and *A. dieffenbachii*, the samples generated by and the protocols used by Che-Pelicier et al. (2025) were followed. The purpose of the Che-Pelicier et al. (2025) study was to compare the eDNA and eRNA decay rates of *A. dieffenbachii* and *A. australis* to assess potential differences between species and eNAs types. Briefly, eNAs were sampled from freshwater tanks with one specimen each of *A. dieffenbachii* and *A. australis* together in a time series after removal of eels from the tanks. Then 100 mL water samples were collected immediately after specimen removal (*t* = 0) and at 4, 8, 12, 18, 24, 72, 120, and 168 h after specimen removal. Multiplex ddPCR with the primers and probes for the NADH5 region designed in this study were used for each of these nine timepoints to assess the degradation rate of eDNA and eRNA of each species. Samples were tested in biological triplicates for each treatment at each time point, and the samples were diluted tenfold to reduce effects of PCR inhibitors and non-specific amplification.

Calculations of initial eNA concentrations as well as decay rate calculations followed those used by Che-Pelicier et al. (2025). Linear mixed models (LMMs) were initially used for assessing significant differences in initial concentrations of eNAs shed in aquaria. Subsequently, general linear mixed effects models (GLMMs) with a gamma distribution and log link were used to assess decay rates. Nucleic acid type and species were fixed effects, and tank replicate was a random effect. Analyses were conducted using lme4 (1.1.37; Bates et al., 2015) and lmerTest (3.1.3; Kuznetsova, Brockhoff & Christensen, 2017) packages. Values of eDNA and eRNA concentrations were log-transformed in the LMM. Significance level was set at 0.05 for all tests. Model selection was based on Akaike Information Criterion (AIC) values. The R package DHARMa (0.4.7; Hartig, 2016) was used to assess the fit of each model by examining residuals. Samples that had no detectable nucleic acid at *t* = 0 were removed from the degradation rate analyses in order to avoid negative decay rates.

## Results

### Primer and probe design

**Figure 2:**
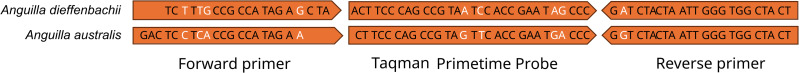
Sequences for species-specific primers and probes to amplify *Anguilla dieffenbachii* and *Anguilla australis*. Base pairs in black indicate conserved areas. Base pairs in white indicate different regions between the species captured by the primers and probes. Both assays are for the NADH5 gene.

**Figure 3:**
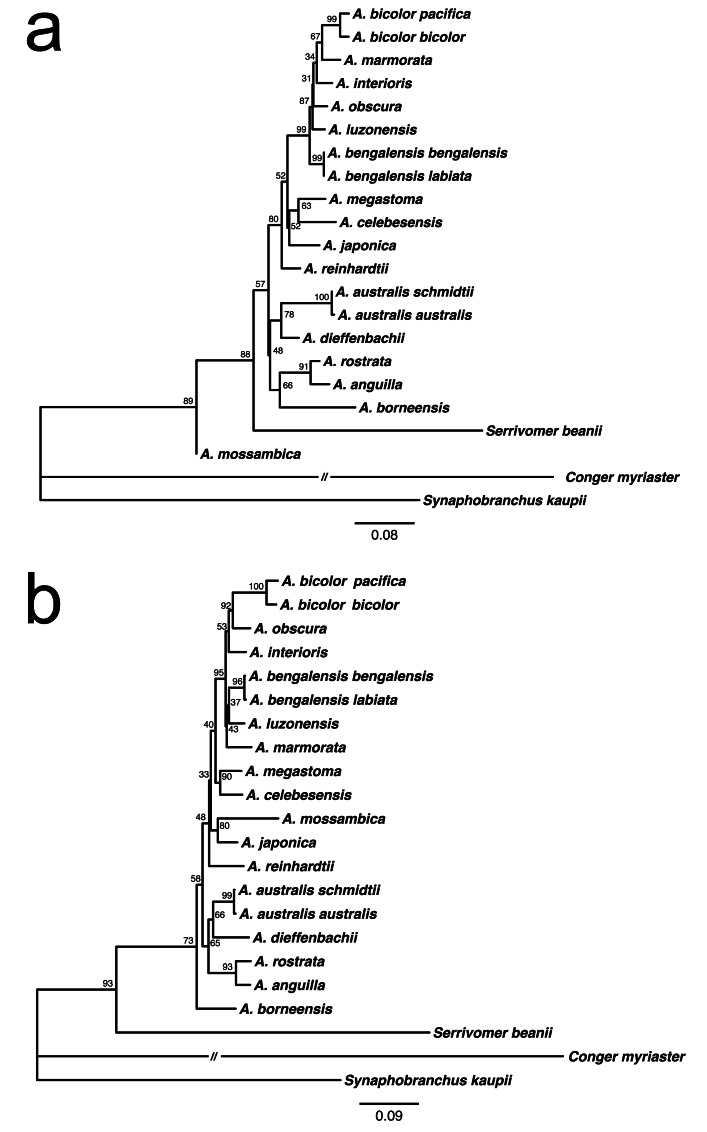
Consensus trees for *Anguilla* species and *Conger myriaster*, *Synaphobranchus kaupii*, and *Serrivomer beanii* as outgroups. Phylogenies were constructed on the (A) NADH5 gene using a TPM2u + F + G4 model and (B) ATP6 gene using a HKY +F + G4 model. Branches shortened to improve visualization are indicated with //. Numbers next to nodes represent bootstrap support (%).

The initial list of in-silico assays was extensive for both target species (*n* = 83 assays for *A. dieffenbachii* and *n* = 62 assays for *A. australis*). These assays targeted a range of mitochondrial genes, tRNAs, and two unspecified sections of the genome. Eleven of the mitochondrial genes included assays designed for both species. *Anguilla dieffenbachii* primers with the ten highest rankings were designed on the displacement loop (D-Loop), NADH5, ATP6, CytB, and COXI genes (Table 2). *Anguilla australis* primers with the ten highest rankings were on the NADH3, NADH4, NADH5, ATP6, and CytB genes (Table 2). Of these, only the NADH5, CytB, and ATP6 were shared between species (Table 2). The assays designed for CytB were not species-specific as the primers had no mismatches between target species and only two mismatches in the probe. The importance of mismatches in primers over probes has been noted elsewhere (Wilcox et al., 2013). Therefore, CytB was not considered a suitable choice for primer and probe design. Multiple assays were designed on the NADH5 gene: three for *A. australis*, and four for *A. dieffenbachii*. The NADH5 assay with the greatest number of base pair mismatches on the primers was chosen for each species. Thus, the chosen NADH5 assay for *A. australis* had five mismatches on the primers and four mismatches in the probe. The NADH5 assay chosen for *A. dieffenbachii* had five mismatches in the primers and one mismatch in the probe (Table 2; Fig. 2). For those on ATP6, there were a total of three mismatches on the primer sets, and zero to one mismatch on the probes. Four sets of primers were ordered for laboratory testing: one set each for *A. dieffenbachii* and *A. australis* on the NADH5 gene, and one set for each species on the ATP6 gene. Sequence similarity for *A. dieffenbachii* and *A. australis* on ATP6 and NADH5 genes was 93% on both genes (Table 2). Phylogenetic inference of the ATP6 and NADH5 genes recovered the two target species as sister taxa, and divergence of the sister target clades from the other Pacific eels included in the phylogenies was moderately supported (Fig. 3). Together, these results indicated that our designed assays could be used to distinguish between the target taxa even in environments where other species of *Anguilla* are found.

**Table 2:** Criteria used to design species-specific primers and probes for *A. australis* and *A. dieffenbachii*. Technique for Order of Preference by Similarity to Ideal Solution (TOPSIS Rank) lists the assays in rank order. Sequence similarity refers to the similarity between the target species and non-target species, which includes eleven other species of *Anguilla* that spawn in the Pacific Ocean. Intraspecific nucleotide diversity refers to intraspecific genetic diversity of the target species. Interspecific nucleotide diversity refers to genetic diversity between the target species and other species of *Anguilla*. Bold font indicates the assays selected for laboratory testing.

**Target species**	**Gene**	**TOPSIS Rank**	**Sequence similarity**	**Intraspecific** ** nucleotide diversity**	**Interspecific nucleotide diversity**	**Percent identity between target species**	**Assay found in both target species?**	**Mismatches between target species in primer regions**	**Mismatches between target species in probe regions**
*A. australis*	**NADH5**	**1**	**0.863**	**0.002**	**0.084**	**93%**	**Yes**	**5**	**4**
	NADH4	2	0.849	0	0.076	93%	No	4	3
	**ATP6**	**3**	**0.874**	**0.005**	**0.065**	**93%**	**Yes**	**3**	**0**
	NADH5	4	0.870	0.002	0.08	93%	Yes	2	4
	CytB	5	0.883	0	0.100	94%	Yes	2	0
	NADH5	6	0.922	0	0.073	93%	Yes	1	2
	CytB	7	0.868	0.005	0.072	94%	Yes	4	0
	NADH3	8	0.891	0.002	0.112	93%	No	1	0
	CytB	9	0.897	0	0.090	94%	Yes	0	2
	CytB	10	0.864	0.012	0.086	94%	Yes	2	2
*A. dieffenbachii*	D-Loop	1	0.759	0.021	0.164	80%	No	3	2
	NADH5	2	0.911	0	0.089	93%	Yes	2	2
	**NADH5**	**3**	**0.851**	**0.005**	**0.124**	**93%**	**Yes**	**5**	**1**
	D-Loop	4	0.903	0	0.085	80%	No	3	3
	CytB	5	0.916	0	0.094	94%	Yes	0	2
	NADH5	6	0.884	0	0.087	93%	Yes	1	2
	COXI	7	0.868	0	0.094	94%	No	3	2
	NADH5	8	0.905	0	0.095	93%	Yes	3	2
	NADH5	9	0.929	0	0.090	93%	Yes	1	4
	**ATP6**	**10**	**0.844**	**0.006**	**0.098**	**93%**	**Yes**	**3**	**1**

### End-point PCR

Positive amplification was detected for both target species at both genes for all annealing temperatures using the primers designed in this study. Some non-specific amplification was detected at annealing temperature up to 60 °C (*A. australis* ATP6 primers amplified *A. celebesensis*, *A. reinhardtii*, and *A. dieffenbachii* tissue; *A. australis* NADH5 primers amplified *A. marmorata*, *A. celebesensis*, *A. megastoma*, *A. japonica*, *A. reinhardtii*, and *A. dieffenbachii* tissue; *A. dieffenbachii* ATP6 primers amplified *A. australis* tissue; and *A. dieffenbachii* NADH5 primers amplified *A. obscura*, *A. australis* tissue) (Table 3).

### Droplet digital PCR species specificity

Droplet digital PCR assays using primers and probes LF_NADH5_F, LF_NADH5_R, LF_NADH5_Probe, SF_NADH5_F, SF_NADH5_R, and SF_NADH5_Probe were tested for two different DNA concentrations (1 ng/µL and 0.1 ng/µL). Assays began at 1 ng/µL in order to standardize against low DNA concentrations of some of the *Anguilla* specimens. The 0.1 ng/µL was included to test the sensitivity of potential cross-reactivity from non-target species. Because no cross-reactivity was detected at 0.1 ng/µL, no further dilutions were tested. These concentrations were used for both the target and non-target *Anguilla* species. Target species *A. dieffenbachii* and *A. australis* were reliably detected at both concentrations in all trials. Some non-target species were only detected at 1 ng/µL in the ddPCR trials (Fig. S1), while no non-target species were detected at 0.1 ng/µL in either the NADH5 or ATP6 assays. The ATP6 assays were not further explored because the *A. australis* ATP6 assay showed high amounts of intermediate fluorescence droplets (“rain”) when tested against *A. dieffenbachii*, *A. reinhardtii* and *A. celebesensis* DNA, and thus downstream analyses focused on NADH5 assays. Some non-target *Anguilla* species showed cross-amplification in end-point PCR, but the inclusion of species-specific probes in the NADH5 ddPCR assay improved both sensitivity and specificity (Fig. S1).

### Limits of detection and limits of quantification

**Figure 4:**
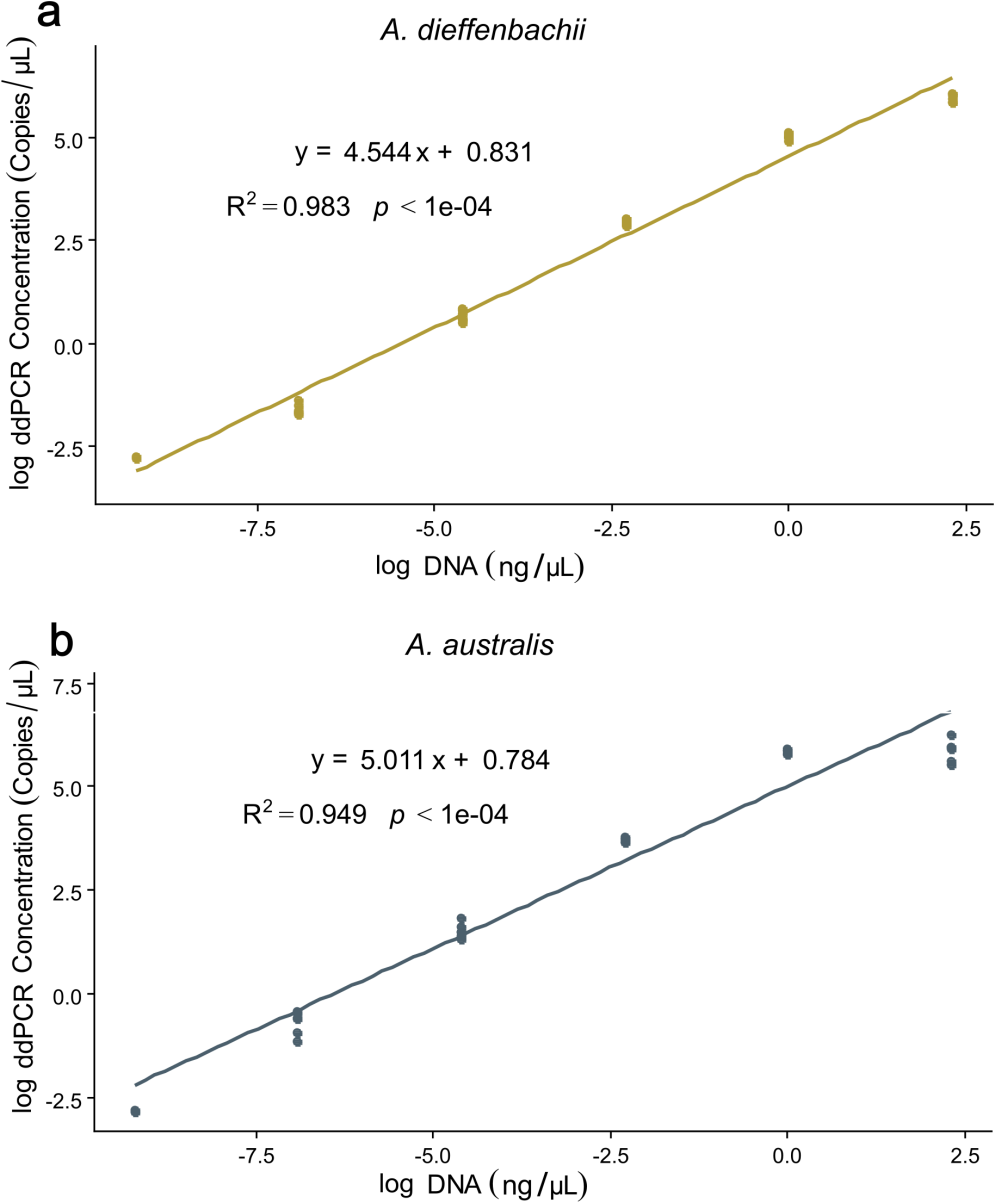
Log linear regressions of *Anguilla dieffenbachii* and *A. australis* analyses for the NADH5 assays. DNA concentration against copy numbers generated by ddPCR: *Anguilla dieffenbachii* (A), and *A. australis* (B). *R*^2^ indicates the goodness-of-fit, and the *p*-value indicates the statistical significance. Note that all loaded DNA concentrations are logarithmic values to enhance visualization.

Limits of detection and quantification were assessed for the NADH5 assays for both species. Concentrations of detected DNA decreased from loading concentrations of 10 ng/µL of DNA of the target species to 0.1 pg/µL, where detection was only recovered in two of six replicates of the assay for each species. There was no detection in any 0.01 pg/µL samples, the lowest template concentration tested. The LOD was 0.20 copies/µL for *A. dieffenbachii* and 0.51 copies/µL for *A. australis*. The LOQ for *A. dieffenbachii* was 0.20 copies/µL, and for *A. australis* is 0.51 copies/µL (Table 4). Detected copy number decreased exponentially with decreasing template concentration. Standard curves were statistically significant for both assays, with strong linear relationships between log transformed copy number and input DNA concentration (*p* < 0.001, *R*^2^ = 0.983 for *A. dieffenbachii*; *p* < 0.001, *R*^2^ = 0.949 for *A. australis*; Fig. 4).

### Quantifications of environmental nucleic acids concentrations

There was no detection in any of the ddPCR NTCs when testing the degradation rate experiment. For both species, eDNA was detected at higher concentrations than eRNA at most timepoints. On average, 4.7 times more DNA was detected from *A. dieffenbachii* than from *A. australis* specimens, and 3.5 times as much RNA was detected from *A. dieffenbachii* specimens as from *A. australis* specimens. However, these differences were not statistically significant (*p* = 0.062, Table S2). Nucleic acid type (*p* < 0.001) did have a significant effect on the concentrations of eNAs detected. For both species, the highest amounts of eDNA were detected at *t* = 4. The highest amounts of eRNA for both species were detected at *t* = 0.

### Degradation rates

The half-life of eDNAs in this study ranged from 5.291 to 23.105 h, and for eRNA from 4.560 to 16.120 h (Table S3). The R^2^ values for decay rates ranged from 0.61 to 0.96, indicating that the models fit the data well. Decay rates *λ* varied from 0.030 to 0.131 for eDNA and from 0.043 to 0.152 for eRNA (Table S3). The decay rate model showed no statistical significance between NA types (*p* = 0.108), or species (*p* = 0.532, Table S2).

## Discussion

### Assay design and species cross-reactivity

In this study, finely-tuned inference methods were used to develop eDNA target assays for two closely-related *Anguilla* species (*A. dieffenbachii* and *A. australis*) whose spawning areas are likely sympatric with other members of their genus. These assays will be used to distinguish *A. dieffenbachii* and *A. australis* from their congenerics in environmental samples, provide further insight as to their species’ distributions and spawning areas, and guide decisions on targeting geographic regions for conservation. Additionally, determining their spawning locations and number of spawning sites can provide useful information for stock assessments. Species-specific primers and probes were designed on the same mitochondrial gene by scanning full mitochondrial genomes of multiple individuals of 13 *Anguilla* species. Both assays were used to successfully amplify their target species in end-point PCR as well as ddPCR tests. The single-gene approach allowed the subsequent quantitative measurement of eDNA and its degradation. Cross-reactivity with non-target species of *Anguilla* was seen with primers alone in end-point PCR. Thus, if using primers and end-point PCR, sequencing the products would strengthen discrimination amongst target and non-target species. Future research on blocking primers and CRISPR assays may be able to aid in blocking amplification of non-target species DNA. In the study herein, the inclusion of probes in the ddPCR trials greatly improved the specificity of the assays.

**Table 3:** Species of *Anguilla* that amplified with species-specific primers in end-point PCR with an annealing temperature of 60 °C. An ‘x’ denotes where amplification was detected with each target species assay. Two microliters of DNA at 1 ng/μ L were loaded into each PCR reaction. No probes were used in end-point PCR.

End-point PCR primer assay	Gene	*A. australis*	*A. dieffenbachii*	*A. celebesensis*	*A. reinhardtii*	*A. marmorata*	*A. megastoma*	*A. japonica*	*A. obscura*
*A. dieffenbachii*	NADH5	x	x						x
	ATP6	x	x						
*A. australis*	NADH5	x		x	x	x	x	x	
	ATP6	x	x	x	x				

**Table 4:** Results of dilution series to assess the limit of detection (LOD) and limit of quantification (LOQ). The LOQ is the mean concentration (copies/μ L) at the last dilution before the coefficient of variance (CV) exceeds 35%. The LOD is the mean concentration (copies/μ L) where the upper 95% confidence interval (Upper 95%) surpasses the mean concentration of the no template control (NTC; loaded DNA of 0 ng/μ L). The LOD and LOQ for each assay are the mean concentration (copies/μ L) in bold.

Species	Loaded DNA (ng/μ L)	Mean concentration (copies/μ L)	CV	Lower 95%	Upper 95%
*A. dieffenbachii*	0	0.01	244%	−0.01	0.03
	1 × 10^−6^	0	NA	0	0
	1 × 10^−5^	0.02	154%	−0.01	0.05
	**1 ×10** ^−4^	**0**.**20**	**12%**	**0**.**18**	**0**.**22**
	1 × 10^−3^	2.01	13%	1.79	2.22
	0.1	19.33	7.03%	18.24	20.42
	1.0	152.17	6.64%	144.08	160.25
	10	392.8	9.64%	362.5	423.1
*A. australis*	0	0	NA	0	0
	1 × 10^−6^	0	NA	0	0
	1 × 10^−5^	0.02	154%	−0.01	0.04
	**1 ×10** ^−4^	**0**.**51**	**26%**	**0**.**40**	**0**.**62**
	1 × 10^−3^	4.65	19%	3.93	5.37
	0.1	40.77	5.26%	39.05	42.48
	1.0	346.3	5.52%	331.02	361.63
	10	360.33	25.78%	286.01	434.65

Sensitivity of the assay is important for eDNA detection, but detecting eNAs also varies based on an organism’s biology. In general, a lower LOD and LOQ indicates greater sensitivity of an assay and is considered preferrable (Klymus et al., 2020). The LOQ and LOD of the assay developed here is comparable to a recently developed goldfish assay, ranging from 0.16 copies/µL to 2.70 copies/µL (Smith et al., 2026). Additionally, our LOQ values were comparable to the LOQ values for four species-specific assays of amberjack and rudderfish (Dias et al., 2025), although the amberjack and rudderfish LOD values were a magnitude more sensitive than the assays generated here. These amberjack and rudderfish assays were also designed on the NADH5 gene (Dias et al., 2025), indicating that this difference in LOD is likely due to differences in the species examined. The loading concentration for assessing the LOD and LOQ for each species of *Anguilla* used in this study was 10^−4^ ng/µL. This is equal to the *A. dieffenbachii* assay and slightly more sensitive than the *A. australis* assay designed by Thomson-Laing et al. (2021), which had 10^−3^ ng/µL as its LOD and LOQ. Although the study herein optimized assay conditions for end-point PCR, there may be different optimal conditions for ddPCR due to partitioning and different enzyme kinetics (Sidstedt, Rådström & Hedman, 2020). Thus, the different LOD and LOQ are likely explained either by potentially different optimal conditions for their respective platforms, or due to examination of different mitochondrial genes, as the study species are the same. Consequently, the assays designed in this study are suitable for detecting target species at low levels of eNA concentrations.

### Environmental NA detection and degradation rates

To accurately compare eRNA between different species, assays should target the same type of RNA. This is because different genes release different amounts of detectable eNAs that persist and decay at different rates. This is evident when comparing our results to that of Che-Pelicier et al. (2025). For example, there was 492 times more eRNA detected on the *A. australis* 16S rRNA assay than the NADH5 assay designed in this study (Che-Pelicier et al., 2025). These discrepancies are most likely due to 16S rRNA being a ribosomal RNA (rRNA), whereas NADH5 and CytB are coding genes. Given that ribosomes are necessary for protein synthesis and are crucial to the life of every known organism (Hori, Engel & Kobayashi, 2023), they are found in cells in high abundances. In contrast, messenger RNA (mRNA) of NADH5 and CytB will only be transcribed when required. This high abundance of rRNA compared to mRNA may be why Che-Pelicier et al. (2025) detected it at such high quantities. Analyzing different types of RNA prevents accurate comparisons between different species and indicates that when comparing quantities of shed eRNA, studies should target the same type of RNA.

Understanding degradation rates of eDNA is crucial, as it may provide information as to how recently the organism was in the environment relative to the time of sampling. Decay rate of eDNA depends on factors such as the ability to form secondary structures, the target fragment length, and source (mitochondrial DNA *vs.* nuclear DNA), which can impact detection and yield of target species DNA (Long & Dawid, 1980; Andres et al., 2023). In the current study, eRNA degraded more quickly than eDNA overall, as has been seen in previous experiments (Scriver et al., 2023), although this does not always hold true in real-life scenarios (Scriver et al., 2024). While eRNA may provide a more time-sensitive signal than eDNA, greater understanding of the ecology of eRNA is needed before making reliable interpretations of its spatiotemporal signal (Jo et al., 2023).

In the phylogenetic inference, *A. dieffenbachii* and the *A. australis* subspecies were resolved as sister taxa for both genes examined in this study (NADH5 and ATP6). In some cases, node support was limited for branches of *Anguilla* species (Fig. 3), which further demonstrates the complexity of creating single-gene assays for organisms within this genus. However, tests with primers and probes designed in the study herein show high sensitivity of the assays. Thus, with phylogenetics alone, we cannot discern species well based on a few base pair mismatches. The high sensitivity of ddPCR is a valuable advancement for making distinctions among sympatric congenerics (Dias et al., 2025).

While the designed species-specific primers and probes in this current study can reliably distinguish NZ *Anguilla* eels from other *Anguilla* species, several challenges remain and should guide future research. First, testing these assays on eNAs derived from saltwater would provide further insight to the effects of environmental conditions on detection and degradation. Previous studies suggest that eDNA degrades more quickly in marine systems than in freshwater systems (Sassoubre et al., 2016), whereas others found that eDNA degradation rates are slower in marine systems (Collins et al., 2018), confounding possible interpretations of how these assays would react in saltwater. Secondly, loading concentrations of DNA above a certain threshold resulted in amplification of non-target species. However, this non-target DNA amplification was far lower than the amplification of target taxa DNA. Concentrations of wild samples of eDNA are not often measured, but when they are, they exhibit a very wide range, from five magnitudes lower to over ten times higher than what was used in the study herein (Lacoursière-Roussel et al., 2016; Vautier et al., 2023). In laboratory experiments, eDNA concentrations can be quite high as well (Takeuchi et al., 2019; Che-Pelicier et al., 2025). In the present study, amplification of target species’ DNA occurred at concentrations as low as 10^−4^ ng/µL, whereas amplification of non-target species’ DNA only occurred at a lower limit of 1 ng/µL. In areas where non-targeted *Anguilla* species may be present and cross-amplification is a concern, additional verification of positive detections could involve sequencing. The possibility of non-specific genetic amplification highlights the difficulty of designing species-specific primers and probes for sympatric species potentially experiencing incomplete reproductive isolation.

## Conclusions

This study presents novel species-specific primers and probes targeting the mitochondrial NADH5 gene for *A. dieffenbachii* and *A. australis* for use in ddPCR of eDNA samples. Testing the assays against the DNA of target and non-target species of *Anguilla*, we found very minimal non-specific amplification only at loading genomic DNA concentrations over 1 ng/uL. Successful amplification of target species at concentrations as low as 10^−4^ ng/uL provides confidence that these assays will detect NZ freshwater eels at such low eDNA concentrations without the risk of detecting other species of *Anguilla*. Genetically distinguishing closely-related sympatric species is difficult, and this study demonstrates how novel bioinformatic tools can aid researchers in overcoming this challenge. Additionally, testing these assays demonstrates why, when assessing eDNA detection limits and degradation rates of different organisms, developing and analyzing assays on the same gene is crucial. Untangling differences in how eDNA and eRNA are detected and degrade from different species is critical to their detection in natural habitats, particularly for taxa that are rare or potentially sympatric. As wild populations of these two species of *Anguilla* are in decline, non-invasive detections with eNAs provide an avenue for understanding biota without disturbing their natural habitat. Ultimately, these tools can be used in the wider Pacific to uncover the unknown migration routes of Aotearoa New Zealand *Anguilla* species, which may be more imperative than ever to conserve these precious creatures in an age of ecological crises.

##  Supplemental Information

10.7717/peerj.21612/supp-1 Supplemental Information 1 Output from ddPCR for degradation experiment of *Anguilla dieffenbachii* and* A. australis* eDNA and eRNA of NADH5 mitochondrial gene assays

10.7717/peerj.21612/supp-2 Supplemental Information 2 Metadata sheet with life-lives and decay rates of eDNA and eRNA of *Anguilla dieffenbachii* and* A. australis* NADH5 mitochondrial gene assays

10.7717/peerj.21612/supp-3 Supplemental Information 3 Output of ddPCR for dilution series of *Anguilla dieffenbachii* and* A. australis* eDNA to calculate limits of detection and quantification of the NADH5 assays

10.7717/peerj.21612/supp-4 Supplemental Information 4 Script for calculating degradation rates of *Anguilla dieffenbachii* and* A. australis* eDNA and eRNA on the NADH5 mitochondrial gene

10.7717/peerj.21612/supp-5 Supplemental Information 5 Script to calculate limits of detection and quantification of the NADH5 assays designed for *Anguilla dieffenbachii* and* A. australis* eDNA

10.7717/peerj.21612/supp-6 Supplemental Information 6 Results from the linear mixed effects model in assessing effects on decay rates of the NADH5 assays designed for *Anguilla dieffenbachii* and *Anguilla australis*

10.7717/peerj.21612/supp-7 Supplemental Information 7 Results from the linear mixed effects model (LMM) effects on decay rates of the NADH5 assays designed for *Anguilla dieffenbachii* and *Anguilla australis*. Significant p-values are indicated in bold.

10.7717/peerj.21612/supp-8 Supplemental Information 8 Metadata table containing information on aquaria for *Anguilla dieffenbachii* and *A. australis* for establishing eNA decay rates decay rates of eDNA, eRNA, and half-lives of eDNA and eRNA.

10.7717/peerj.21612/supp-9 Supplemental Information 9 Statistics from the generalized linear mixed effects model (GLMM) The GLMM was used to calculate decay rates of eDNA and eRNA of *A. dieffenbachii* and *A. australis*.

10.7717/peerj.21612/supp-10 Supplemental Information 10 Contributions of random effects from the decay rate generalized linear model (GLMM) Decay rates were evaluated using a Gamma model with a log link. Aquarium ID was included as a random effect to account for differences in eNA detections between aquaria.

10.7717/peerj.21612/supp-11 Supplemental Information 11 Contributions of fixed effects from the decay rate generalised linear mixed effects model (GLMM) Decay rates were evaluated using a Gamma model and log link. Significant p-values are indicated in bold.

10.7717/peerj.21612/supp-12 Supplemental Information 12 Test for contribution of random effects by comparing a generalized linear model (GLM) and generalized linear mixed-effects model (GLMM) Aquarium ID was considered a random effect to account for potential variation between aquaria that may influence eNA concentrations. Significant p-values are indicated in bold.

10.7717/peerj.21612/supp-13 Supplemental Information 13 Species of *Anguilla* that amplified with species-specific primers and probes in ddPCR Only the species of *Anguilla* whose DNA amplified are shown below. The eDNA controls were generated by Che-Pelicier et al. (2025). Both plots shown use the NADH5 assays. (a) displays species detected using *A. dieffenbachii* primers and probes, (b) displays species detected using *A. australis* primers and probes. All assays produced non-specific amplification with loaded DNA concentrations of 1 ng/µL, while non-specific amplification was not detected at 0.1 ng/µL.

## Additional Information and Declarations

### Data Availability

The following information was supplied regarding data availability: The data and code are available in the Supplemental Files.
