# Supplementary material for: From rivers to the sea: a single gene assay for New Zealand freshwater eel eDNA monitoring
Source: PeerJ. 2026 Aug 5;14:e21612. doi: 10.7717/peerj.21612 (PMC13452424; doi:10.7717/peerj.21612)
Supplement: Supplementary file 1 [file peerj-14-21612-s007.docx]

**Supplemental Table 2.** Results from the linear mixed effects model (LMM) in assessing effects on decay rates of the NADH5 assays designed for *Anguilla dieffenbachii* and *Anguilla australis*. Significant p-values are indicated in bold.

| **Initial Concentrations** | | |
| --- | --- | --- |
| Fixed Effect | t | p |
| Intercept | -1.705 | 0.132 |
| biomass | 1.938 | 0.093 |
| NA type | 6.557 | **0.000317** |
| Species | -2.22 | 0.062 |
| NA type:Species | 0.097 | 0.926 |
|  | | |
