# Supplementary material for: From rivers to the sea: a single gene assay for New Zealand freshwater eel eDNA monitoring
Source: PeerJ. 2026 Aug 5;14:e21612. doi: 10.7717/peerj.21612 (PMC13452424; doi:10.7717/peerj.21612)
Supplement: Supplementary file 2 [file peerj-14-21612-s009.docx]

**Supplemental Table 4.** Statistics from the generalized linear mixed effects model (GLMM) to calculate decay rates of eDNA and eRNA of *A. dieffenbachii* and *A. australis*.

| **Model decay statistics:** | | | | |
| --- | --- | --- | --- | --- |
| AIC | BIC | logLik | -2*log(L) | df.resid |
| -39.0 | -36.6 | 24.5 | -49.0 | 7 |
| Scaled residuals: | | | | |
| Min | 1Q | Median | 3Q | Max |
| -1.6215 | -0.5687 | -0.1308 | 0.7202 | 1.3140 |
