# Supplementary material for: From rivers to the sea: a single gene assay for New Zealand freshwater eel eDNA monitoring
Source: PeerJ. 2026 Aug 5;14:e21612. doi: 10.7717/peerj.21612 (PMC13452424; doi:10.7717/peerj.21612)
Supplement: Supplementary file 3 [file peerj-14-21612-s010.docx]

**Supplemental Table 5.** Contributions of random effects from the decay rate GLMM.

| **Model decay random effects** | | | |
| --- | --- | --- | --- |
| Number of observations | Groups | Variance | St. Dev |
| 12 | 3 | 0.5595 | 0.748 |
