# Supplementary material for: From rivers to the sea: a single gene assay for New Zealand freshwater eel eDNA monitoring
Source: PeerJ. 2026 Aug 5;14:e21612. doi: 10.7717/peerj.21612 (PMC13452424; doi:10.7717/peerj.21612)
Supplement: Supplementary file 4 [file peerj-14-21612-s011.docx]

**Supplemental Table 6.** Contributions of fixed effects from the decay rate GLMM. Significant p-values are indicated in bold.

| **Model decay fixed effects** | | |
| --- | --- | --- |
| Family: Gamma (log) | | |
| Formula: decay_rate ~ NA_type + species + (1 \| aquarium_longfin_shortfin_control) | | |
| Fixed Effect | t | p |
| Intercept | -7.775 | **7.57e-15** |
| NA type | -1.609 | 0.108 |
| Species | 0.625 | 0.532 |
