# Supplementary material for: From rivers to the sea: a single gene assay for New Zealand freshwater eel eDNA monitoring
Source: PeerJ. 2026 Aug 5;14:e21612. doi: 10.7717/peerj.21612 (PMC13452424; doi:10.7717/peerj.21612)
Supplement: Supplementary file 5 [file peerj-14-21612-s012.docx]

**Supplemental Table 7.** Test for contribution of random effects by comparing a generalized linear model (GLM) and generalized linear mixed-effects model (GLMM). Aquarium ID was considered a random effect to account for potential variation between aquaria that may influence eNA concentrations. Significant p-values are indicated in bold.

| **Significance test between GLM and GLMER** | | | | | | | | |
| --- | --- | --- | --- | --- | --- | --- | --- | --- |
|  | npar | AIC | BIC | logLik | -2*log(L) | Chisq | Df | p |
| glm | 4 | -36.203 | -34.263 | 22.101 | -44.203 |  |  |  |
| glmer | 5 | -39.017 | -36.592 | 24.508 | -49.017 | 4.8141 | 1 | **0.02823** |
